# Retraction Note: Effect of norepinephrine and phenylephrine on prothrombotic response in patients undergoing cesarean section under spinal anesthesia: protocol for a randomized, double-blind, controlled study

**DOI:** 10.1186/s13063-026-09851-9

**Published:** 2026-06-09

**Authors:** Wenhui Tao, Yufang Xie, Wei Ding, Jinfeng Bao, Ye Zhang, Xianwen Hu

**Affiliations:** 1 Department of Anesthesiology, The Second Affiliated Hospital of Anhui Medical University, Hefei City, Anhui Province China; 2 Key Laboratory of Anesthesiology and Perioperative Medicine of Anhui Higher Education Institutes, Anhui Medical University, Hefei City, Anhui Province China; 3 Department of Anesthesiology, The Second People’s Hospital of Hefei, Hefei Hospital Affiliated to Anhui Medical University, Hefei, Anhui 230011 China

**Retraction Note: Trials 25, 432 (2024)**

**https://doi.org/10.1186/s13063-024-08255-x**

The Editor-in-Chief has retracted this article.

After publication, concerns were raised about the trial registration in this article. Post-publication review found the sample size, primary outcome, and secondary outcomes in this article are inconsistent with the registered trial. In addition, the change records of the registered trial lack clarification and transparency, and do not include the version of this article or a clear mapping between them.

Author Wenhui Tao has stated that all authors disagree with this retraction.

